# The CD2–CD58 axis: A novel marker predicting poor prognosis in patients with low‐grade gliomas and potential therapeutic approaches

**DOI:** 10.1002/iid3.1022

**Published:** 2023-10-13

**Authors:** Mingwei Wu, Yiyuan Chen, Gao Hua, Liu Chunhui

**Affiliations:** ^1^ Qinzhou First People's Hospital Qinzhou China; ^2^ Beijing Tiantan Hospital Capital Medical University Beijing China

**Keywords:** CD2, CD58, immune infiltration, inhibitory immune checkpoint, low‐grade glioma, prognosis

## Abstract

**Introduction:**

Low‐grade gliomas (LGGs) are currently considered a premalignant condition for high‐grade gliomas (HGGs) and are characterized by a relatively intact immune system. Immunotherapeutic modalities may offer a safe and effective treatment option for these patients. *However*, the CD2–CD58 axis, an important component of the immunological synapse, remains unknown in LGG.

**Methods:**

RNA‐seq data from TCGA databases were analyzed. Immune cell infiltration was determined using a single‐sample gene set enrichment analysis (ssGSEA) based on integrated immune gene sets from published studies. Kaplan–Meier survival analysis, univariate and multivariate logistic analysis, and the ESTIMATE algorithm were employed to evaluate the impact of the CD2–CD58 axis on adult LGG patients.

**Results:**

The expression of the CD2–CD58 axis was found to be elevated with increasing of WHO grade (*p* < .05). Uni‐ and multi‐variable logistic analysis demonstrated that age, WHO grade, and CD58 levels were associated with poor prognosis in LGG patients with (*p* < .01). MetaSape pathways analysis revealed the involvement of CD58 in regulating T cell activation, leukocyte‐mediated immunity, and the positive regulation of cell activation in WHO grade II and III. CD58 expression correlated with infiltrations of CD4+ lymphocytes, NK cells, and macrophages cells. The ESTIMATE algorithm indicated that patients with high CD58 expression had significantly higher immune scores compared with low CD58 expression in WHO grade II/III, but no statistical difference was observed in WHO grade IV (*p* < .05). Furthermore, correlation analysis demonstrated the significant association between CD58 and CD274 (*r* = 0.581, *p* < .001), HAVCR2 (*r* = 0.58i7, *p* < .001), and LGALS9 (*r* = 0.566, *p* < .001). Immunohistochemical staining further confirmed the relationship of CD58, HAVCR2, WHO grade, and prognosis in grade II and III patients.

**Conclusion:**

Overall, our findings highlight the significant association between the CD2–CD58 axis and poor survival in LGG patients. High CD58 expression is implicated in T cell‐mediated immune responses as an immunosuppressive factor and affect inhibitory immune checkpoint genes.

## Graphical abstract

CD2‐CD58 axis is notably related to poor survival of LGGs, and high CD58 expression is involved in T cell‐mediated immune responses as an immunosuppressive factor by affecting inhibitory immune checkpoint genes.

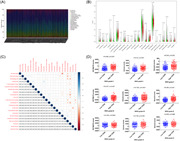

## 1 INTRODUCTION

Gliomas are the most common type of intracranial tumors, and low‐grade gliomas typically refer to WHO grade I and II gliomas, according to conventional histopathology, which account for 6% of central nerve system primary tumors in adults.^1^, ^2^ Histological distinction alone does not significantly impact current clinical treatment.^3^ However, molecular subtypes play a significant role in treatment and prognosis, rather than histological distinction. Genome‐wide molecular‐profiling studies have revealed the characteristic genetic alterations and epigenetic profiles of gliomas and the WHO Classification of Tumors of the Central Nervous System incorporated molecular biomarkers, which are used to refine glioma classification, are able to predict patient outcomes and guide individualized treatments.^3^, ^4^ Molecular pathology and clinical characteristics impact survival in patients with glioma, and extensive resection increased survival in specific patients with IDH‐mutated astrocytoma.^5^

Conventional therapies, such as surgery, chemotherapy, and radiotherapy, have shown limited improvements in the complete remission and overall survival (OS) of glioma patients.^6^ In the decades before that, few drugs other than temozolomide had been approved by the Food and Drug Administration, despite numerous clinical trials for glioma. The RAS/MAPK pathway, growth factor receptors, neurotrophic tyrosine receptor kinase, cell cycle signaling, and altered genomic stability are the candidate targets in European Association of Neuro‐Oncology guidelines.^7^ Recently, immunotherapy, a revolutionary approach in cancer treatment, has emerged as a promising strategy in cancer treatment, as it can penetrate the blood–brain barrier and enhance both local and systemic antitumor immune responses.^3^ Given that the neoadjuvant administration of the PDL1/PD‐1 blockade enhances the antitumor immune response of the local and whole body, immunotherapy currently holds a leading position in cancer care.^8^, ^9^

CD58, also known as lymphocyte function‐associated antigen‐3, is a cell‐surface protein and a cell adhesion molecule belonging to the immunoglobulin superfamily.^10^ As the natural ligand of CD58, CD2 is primarily expressed on the surface of T lymphocyte cell and natural killer (NK) cells.^11^ The interaction between CD2 and CD58 plays a crucial role in the costimulatory signal for optimal T cell activation and the modulation of clinical antitumor T cell responses.^8^, ^9^, ^10^ The expression levels of CD58 are closely correlated with the levels of immune‐related markers in pancreatic ductal adenocarcinoma.^11^ Additionally, studies using CD58 knockout mouse models have demonstrated reduced cytokine production and the loss of tumor‐killing ability in CAR T cells.^12^ Given that CD58 stimulates and enhances T cell receptor signaling by engaging CD2, the CD58 locus is an attractive target molecule for understanding immune system dysfunction associated with LGGs due to its involvement in T cell receptor signaling.

The standard of care for LGG patients includes maximal safe resection, followed by dynamic MRI image monitoring in low‐risk patients, and standard radiation and chemotherapy in high‐risk patients.^13^ In this study, we aimed to explore the relationship between CD58 and the prognosis of adult LGG patients using the TCGA database. The tumor immune estimation resource (TIMER) algorithm was further employed to assess CD58 as an independent prognostic factor for OS in LGG patients.

## 2 MATERIAL AND METHODS

### 2.1 Data source, pre‐processing, and immune evaluation

A total of 609 expression profiles were acquired from the TCGA database (http://www.tcga.org/). The dataset included 216 grade II, 241 grade III, and 152 grade IV patients. Two patients were excluded due to age (<18 years). Raw RNA‐seq data were normalized using the LIMMA package in the R platform. The infiltration cell types and the levels of immune cell were determined using Cibersort with default parameters.

### 2.2 Survival analysis

Normalized mRNA gene levels of CD2, CD48, CD58, CD59, and clinical outcomes were used for survival analysis. Patients were categorized into high and low expression groups based on the median value of genes. Multivariate logistic regressive analysis evaluated the prognostic values of CD2, CD48, CD59, and CD59 using the survival package in the R platform (https://cran.r-project.org/web/packages/survival/index.html). The low expression group for CD2, CD48, CD58, and CD59 was used as the reference, with adjustment for tumor stage. A P‐value of <0.05 indicated a statistically significant difference, designating the respective gene as a prognostic gene.

### 2.3 Differential expressed gene (DEG) analysis and gene set enrichment analysis (GSEA)

The LIMMA package was used for differential analysis, and the GO and KEEG analyses of DEGs were performed using the clusterProfiler and enrichplot packages (https://github.com/GuangchuangYu/enrichplot). Additionally, GSEA (http://software.broadinstitute.org/gsea/index.jsp) was conducted to assess the biological pathways associated with the prognostic CD2–CD58 axis. GSEA utilized the Molecular Signatures Database (MSigDB, http://software.broadinstitute.org/gsea/msigdb/index.jsp) c2 (c2. all.v7.4. symbols. gmt).

### 2.4 Immunocytes infiltration analysis

The “GSVA” package was used for single‐sample GSEA (ssGSEA) to quantify immune cell infiltration kinds and levels based on the TPM values of each sample. The R package “ESTIMATE” was used to evaluate the immune score, stromal score, and ESTIMATEScore of different groups. Moreover, Spearman's correlation analysis was conducted to evaluate the correlations between the 28 immune cell types in tumor samples.

### 2.5 Immunochemistry staining

The study included 32 patients (17 males, 15 females) enrolled at Qinzhou First People's Hospital from February 2010 to August 2020. The study protocols adhered to the ethical guidelines of the Declaration of Helsinki and were approved by the Internal Review Board of Qinzhou First People's Hospital. Tissue sections of 4‐μm were incubated with primary antibodies against CD58 (mouse monoclonal, 1:2500, ab238566, Abcam), HAVCR2 (rabbit monoclonal, 1:500, ab241332, Abcam), and LGALS9 (rabbit monoclonal, 1:400, ab227046, Abcam). An H‐score was calculated based on the staining intensity and positive ratio, ranging from 0 to 12.

### 2.6 Statistical analysis

Statistical analyses were conducted using SPSS version 19.0 software (IBM Corp.) and R3.3.1 (https://www.r-project.org). The median TPM value of the CD2–CD58 axis served as the cutoff value to separate patients into the high and low groups. Survival analyses were performed using the Kaplan–Meier method and the Cox proportional hazard regression model. A value of *p* < .05 was considered to indicate a statistically significant difference.

## 3 RESULTS

### 3.1 Clinical and pathological features of glioma patients from TCGA database

A total of 607 glioma patients were included in the transcriptomic analysis, consisting of 352 males and 255 females, with average age of 47.43 ± 0.62 years (range: 18–89 years). The analysis revealed a correlation between the WHO classification and various clinical and pathological features. Elderly patients had a higher representation of higher WHO grades, along with increased death incidence and wild‐type IDH status in Table 1. Additionally, non‐CODEL 1p19q deletion was more prevalent in advanced WHO grades and a shorter OS was observed (*p* < .001). However, there was no statistical difference regarding sex among patients with grades II–IV (*p* = .14).

**Table 1:** Clinical–pathological features of 597 patients with glioma.

Variable	LGG		GBM (WHO IV)	*p*‐value
	WHO II	WHO III		
**Sex**				.14
Male	116	138	98	
Female	98	103	54	
**Age** (year, mean ± sem)	40.66 ± 0.89	45.61 ± 0.86	59.86 ± 1.10	<.001
**OS** (year, mean ± sem)	2.28 ± 0.18	1.81 ± 0.15	0.96 ± 0.07	<.001
**IHD**				<.001
Mutant	193	174	9	
Wild‐type	19	67	139	
NA	2	0	4	
**1p19q**				<.001
CODEL	50	70	0	
Non‐CODEL	134	171	147	
NA	0	0	5	
**CD2** (TPM value)	6.48 ± 0.54	12.71 ± 0.95	23.05 ± 1.25	<.001
**CD48** (TPM value)	9.50 ± 0.78	13.16 ± 0.80	321.17 ± 1.35	<.001
**CD58** (TPM value)	31.77 ± 0.88	40.06 ± 1.21	70.76 ± 1.31	<.001
**CD59** (TPM value)	124.7 ± 0.87	121.6 ± 0.87	124 ± 1.31	.048

### 3.2 Survival analysis of the CD2–CD58 axis in LGG patients

**Figure 1:**
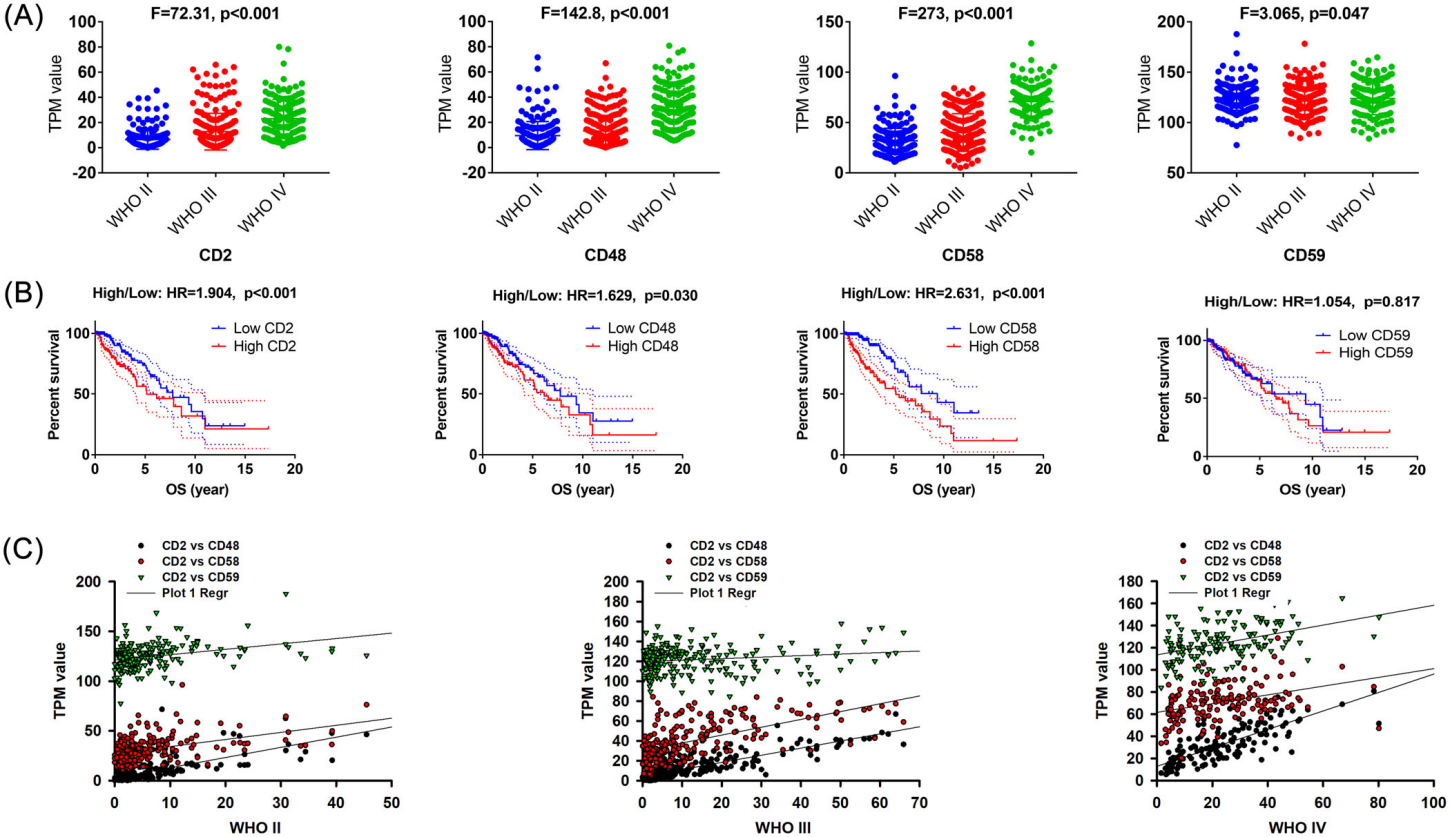
The CD2‐CD58 axis in glioma. (A) The expression levels of CD2‐CD58 axis based on WHO grades. (B) The relationship of CD2‐CD58 with OS in glioma. (C) Pearson correlation analysis showed that the internal relatedness of CD2‐CD58 axis.

The expression levels of CD2, CD48, and CD58 were the lowest in the WHO II gliomas, and the highest in the WHO IV group, as shown in Table 1 and Figure 1A (*p* < .001), while there was no significant difference in CD59 expression between the WHO II–IV groups. Gene expression profiling interactive analysis (GEPIA) revealed that CD2, CD48, and CD58 were associated with the poor OS in LGG patients in Figure 1B. CD58 showed the highest hazard ratio in LGG patients (HR = 2.631, *p* < .001), followed by CD2 (HR = 1.904, *p* < .001) and CD48 (HR = 1.629, *p* = .030). Pearson's correlation analysis demonstrated the positive correlation between CD2 and CD48 in WHO II (*r* = 0.710, *p* < .001), WHO III (0.846, *p* < .001), and WHO IV (0.337, *p* < .001). Similarly, CD2 showed a positive correlation with CD58 in WHO II (*r* = 0.439, *p* < .001), WHO III (*r* = 0.615, *p* < .001), and WHO IV (*r* = 0.377, *p* < .001) gliomas in Figure 1C. Uni‐variable logistic regression analyses identified age, WHO grade, 1p19q_codel status, and CD59 levels as independent risk factors for poor prognosis in LGG patients. Multivariable analysis further revealed age (HR = 1.048, *p* < .001), WHO grade (HR = 2.299, *p* < .001), and CD58 levels (HR = 1.369, *p* < .001) as independent risk factors for poor prognosis, as seen in in Table 2.

**Table 2:** Uni‐ and multi‐variable logistic analysis of factors associated with poor prognosis in patients with LGG.

	Univariate analysis		Multivariate analysis	
	HR (95%CI)	*p* value	OR (95%CI)	*p* value
Age	1.054 (1.030–1.079)	.000	1.048 (1.026–1.070)	.000
Gender	1.070 (0.619–1.851)	.808	‐‐	‐‐
WHO Grade	2.512 (1.316–4.797)	.005	2.299 (1.239–4.267)	.008
IHD‐mutation	1.088 (0.403–2.934)	.868	‐‐	‐‐
1p19q_codel	2.267 (1.014–5.070)	.046	‐‐	‐‐
*CD2*	0.999 (0.964–1.036)	.978	‐‐	‐‐
*CD48*	1.009 (0.971–1.047)	.653	‐‐	‐‐
*CD58*	1.019 (0.989–1.050)	.213	1.369 (1.233–1.521)	.000
*CD59*	1.008 (1.316–4.797)	.005	‐‐	‐‐

### 3.3 Differentially expressed gene (DEG) enrichment analysis based on CD58 levels

**Figure 2:**
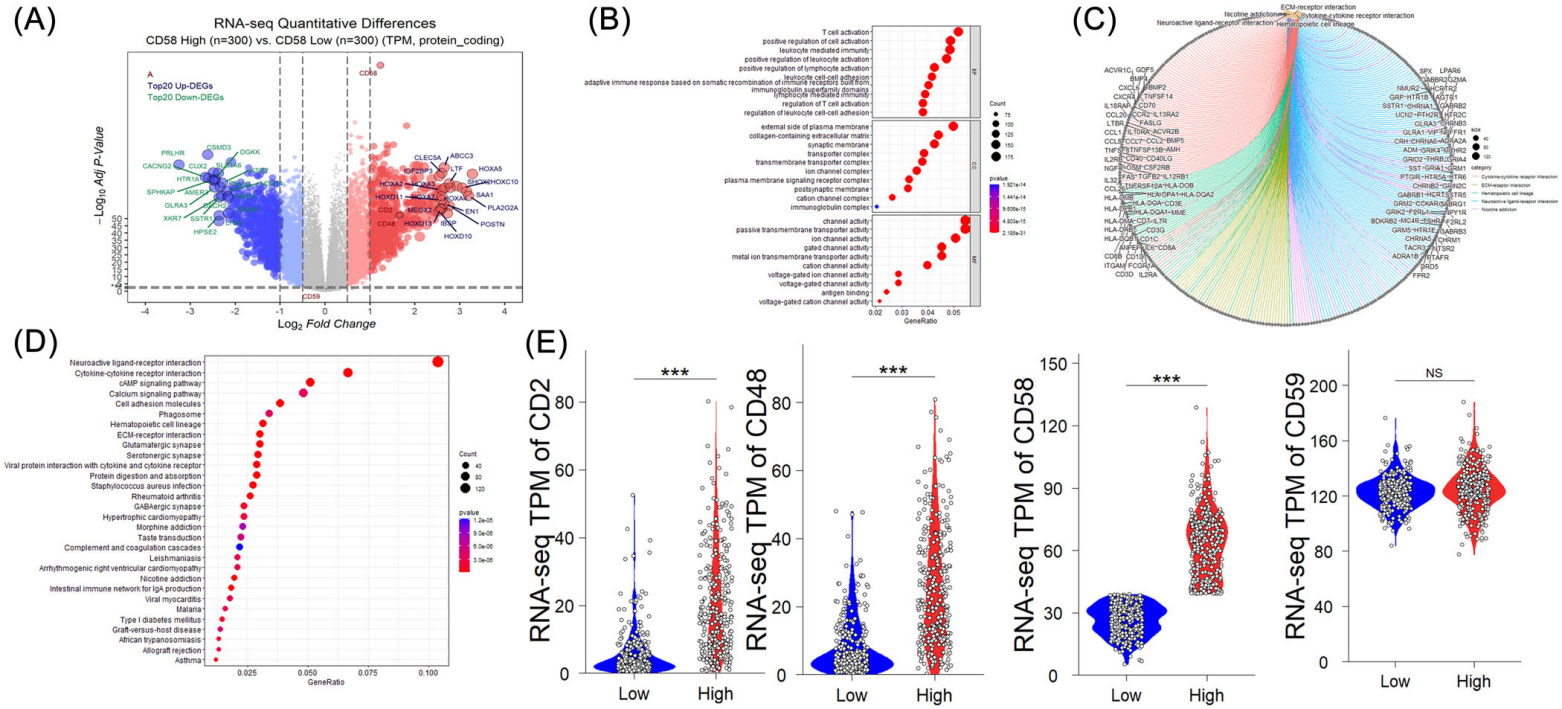
Differential gene‐enrichment analyses of different CD58 groups in 597 patients. (A) Volcano map of differentially expressed genes. **p < .01, ***p < .001. (B) The most enriched GO terms. (C, D) KEGG pathway analysis based on differentially expressed proteins. (E) The expression levels of CD2‐CD58 axis between high and low CD58 groups.

**Figure 3:**
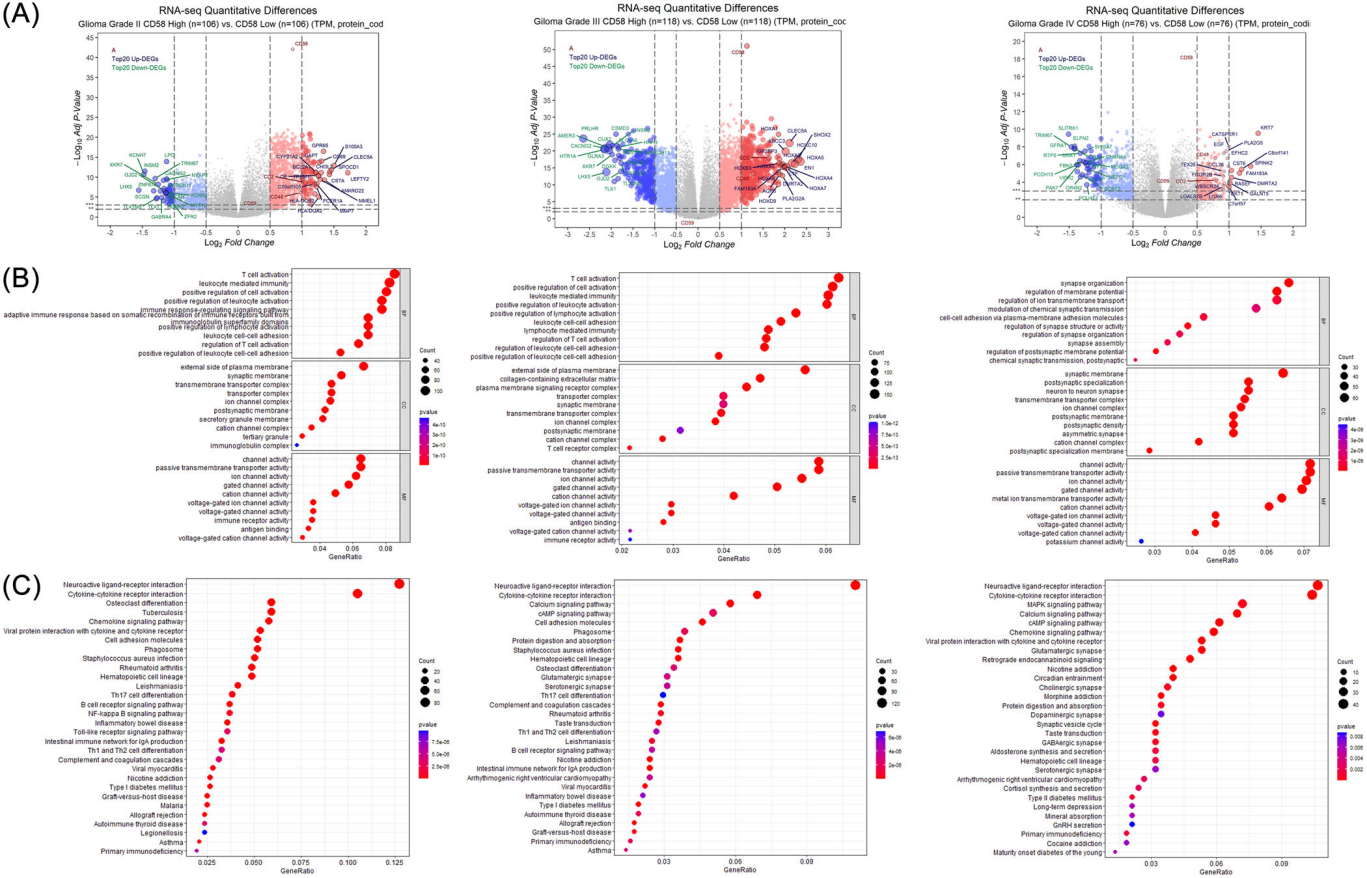
Differential gene‐enrichment analyses of different CD58 groups among WHO grade II, III, and IV. (A) Volcano map of differentially expressed proteins. The most enriched GO terms. (C) KEGG pathway analysis.

Based on the median mRNA level of CD58, the patients were divided into high‐ and low‐CD58 groups (*n* = 597). Differential analysis identified 81 upregulated genes and 61 downregulated genes in the high CD58 group compared to the low CD58 group ( | log2FC | >2, adjusted *p* value < .001), as seen in Figure 2A. Enrichment analysis using the Metascape database revealed that the enriched gene ontology (GO) terms in the high CD58 group were related to T cell activation, the positive regulation of cell activation, and leukocyte‐mediated immunity, as shown in Figure 2B. The top three pathways were the neuroactive ligand–receptor interactions, the cytokine–cytokine receptor interactions, and the cAMP signaling pathway, as shown in Figure 2C–D. The further analysis of DEGs in the high and low CD58 groups within WHO grade II, III, and IV gliomas showed that the differences in the CD2 and CD48 expression between WHO II and III were more pronounced compared to WHO IV, as seen in Figure 3A. The top three GO terms in WHO II and III were T cell activation, leukocyte‐mediated immunity, and the positive regulation of cell activation, while in WHO IV, the top three were synapse organization, the regulation of membrane potential, and the regulation of ion transmembrane transport, as shown in Figure 3B. There were no remarkable differences in the top three pathways among the three groups, as seen in Figure 3C.

### 3.4 Immunologic signatures of LGG patients

**Figure 4:**
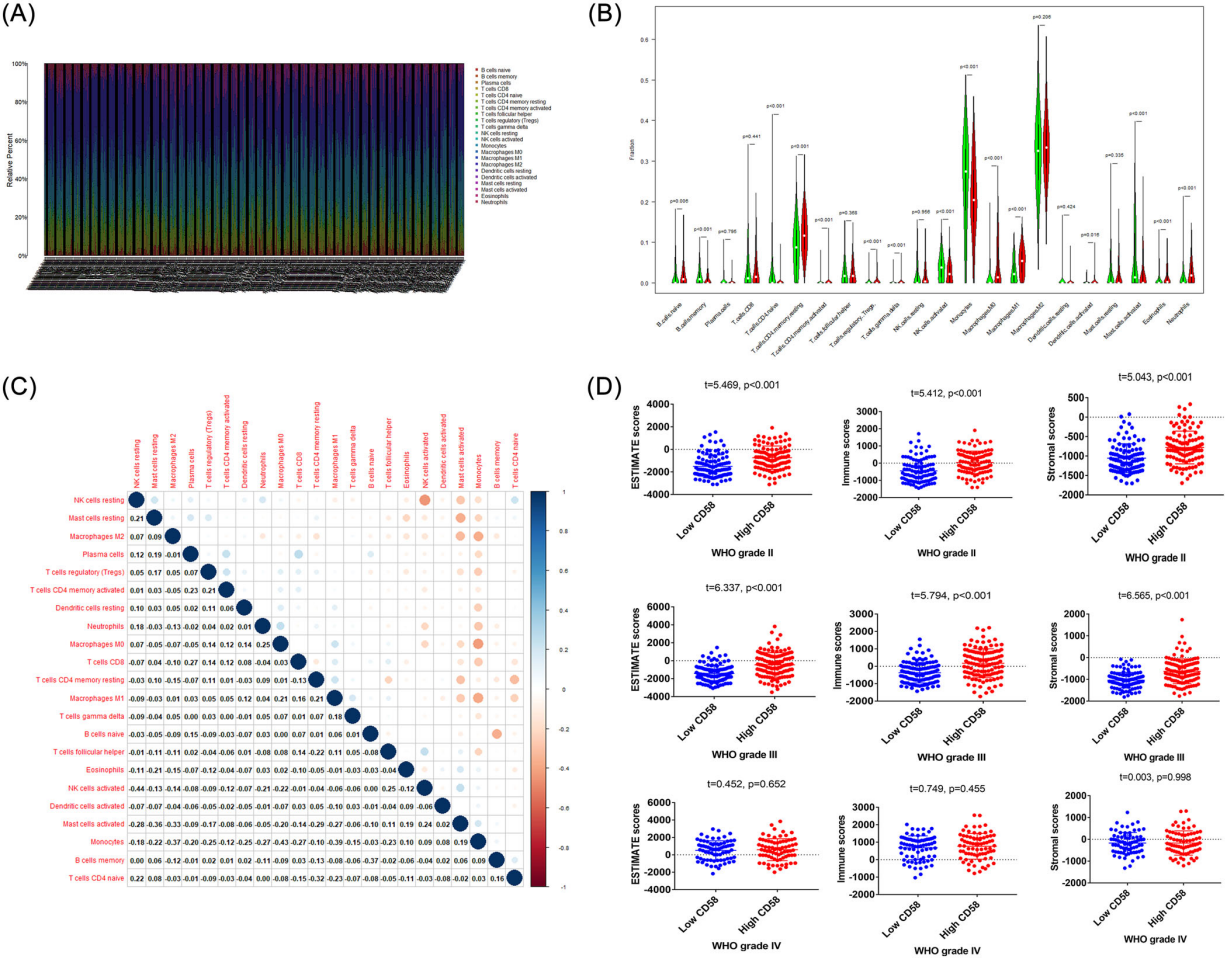
The immune landscape in patient with LGG. (A) Heatmap from immune‐cell signatures in patients with LGG. (B) All 22 subtypes of tumor‐infiltrating immune cells in patients with a high CD58 compared with patients with a low CD58. The following conventions for symbols indicate statistical significance: blank: *p* > .05; *: *p* < .05; **: *p* < .01. (C) Heatmap showing that the ratios of the different tumor infiltration immune cell subgroups were moderately to strongly correlated. (D) Immune scores of patients with LGG according to ESTIMATE based on WHO grades.

A heatmap analysis was performed to uncover the types and ratios of immune cells in LGG patients, as shown in Figure 4A. There were significant difference in CD4+ lymphocytes, NK cells, and macrophages between high CD58 and low CD58 groups (*p* < .05), as shown in Figure 4B. The correlation analysis of 22 immune cell types revealed moderate to strong correlations between different tumor‐infiltrating immune cell subgroups, as shown in Figure 4C. Additionally, significant differences were observed in stromal score, immune score, and ESTIMATEScore between high CD58 and low CD58 groups in WHO grade II and III gliomas (*p* < .001), while no difference was observed in WHO grade IV gliomas (*p* > .05), as shown in Figure 4D.

### 3.5 Relationship between CD58 and immune checkpoint genes in LGG patients

**Figure 5:**
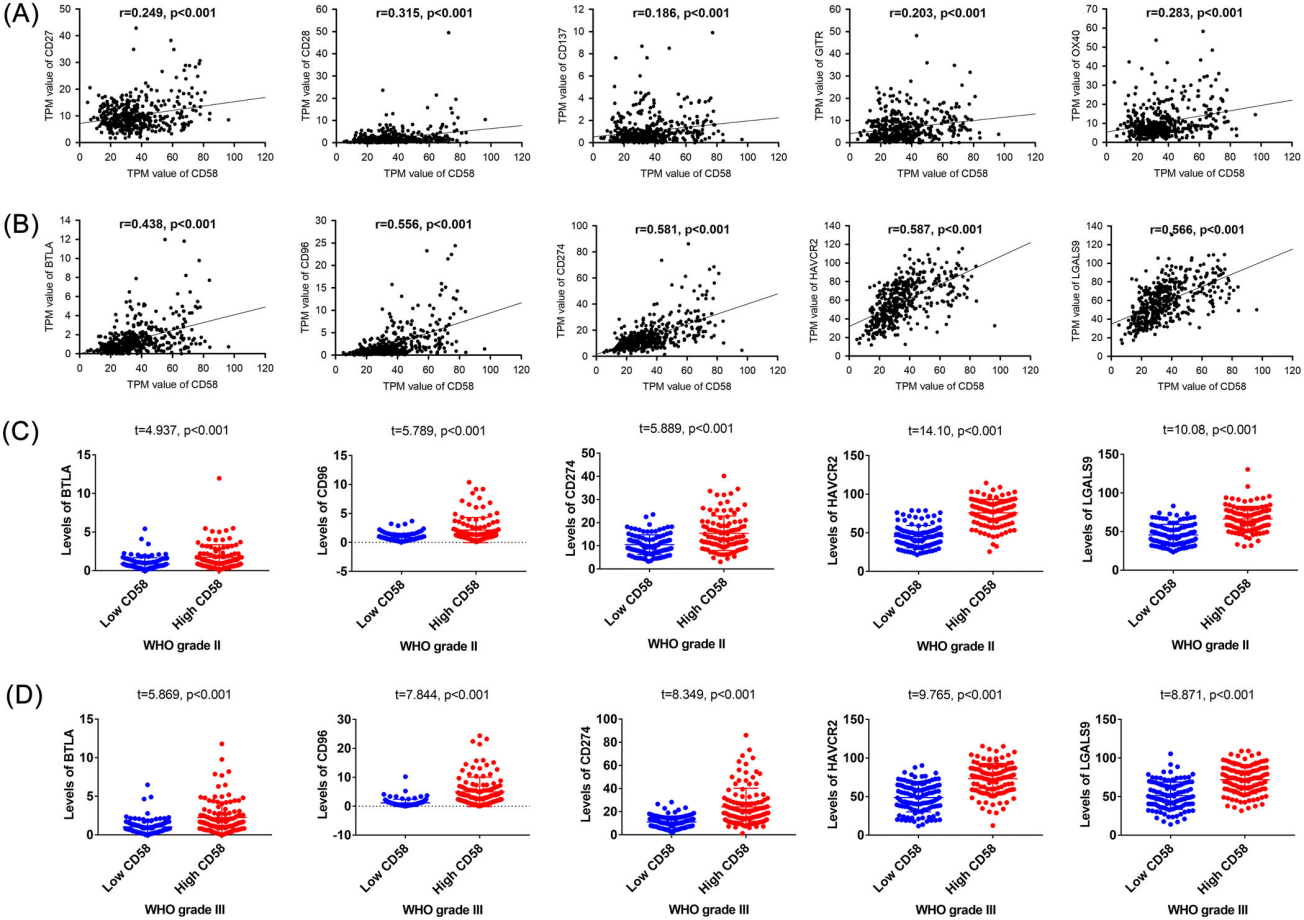
The expression profile of immune checkpoint genes in patients with LGG. (A) The correlation relationship of stimulatory immune checkpoint genes with CD58 in patients with LGG. (B) The correlation relationship of inhibitory immune checkpoint genes with CD58 in patients with LGG. (C) The expression levels of inhibitory immune checkpoint genes in WHO grade II. (D) The expression levels of inhibitory immune checkpoint genes in WHO grade III.

We examined the correlation between CD58 and stimulatory or inhibitory immune checkpoint genes in LGG patients, as shown in Figure 5A–B. CD58 showed positive correlations with stimulatory checkpoint genes, including CD27, CD28, CD137, GITR, and OX40, with correlation coefficients ranging from 0.186 to 0.315. In contrast, CD58 exhibited positive correlations with inhibitory checkpoint genes, including BTLA, CD96, CD274, HAVCR2, and LGALS9, with correlation coefficients ranging from 0.249 to 0.587. Furthermore, the expression levels of inhibitory checkpoint genes were significantly higher in the high CD58 group compared to the low CD58 group in LGG patients, as shown in Figure 5C–D (*p* < .001). Uni‐ and multivariable logistic regression analyses identified HAVCR2 and LGALS9 as independent risk factors for poor prognosis in LGG patients in Table 3.

**Table 3:** Uni‐ and multi‐variable logistic analysis of inhibitory checkpoint genes associated with poor prognosis in patients with LGG.

	Univariate analysis		Multivariate analysis	
	HR (95%CI)	*P*‐value	OR (95% CI)	*P*‐value
Age	1.042 (1.02–1.079)	0	1.04 (1.018–1.063)	0
Gender	1.014 (0.596–1.726)	.968	‐‐	‐‐
WHO Grade	2.033 (1.132–3.652)	.018	2.059 (1.156–3.667)	.014
IHD‐mutation	1.466 (0.707–3.04)	.3048	‐‐	‐‐
1p19q_codel	1.604 (0.708–3.631)	.257	‐‐	‐‐
*BTLA*	1.002 (0.829–1.212)	.983		
*CD96*	1.037 (0.94–1.144)	.464	‐‐	‐‐
*CD274*	0.998 (0.97–1.026)	.866	‐‐	‐‐
*HAVCR2*	1.057 (1.021–1.095)	.002	1.066 (1.033–1.1)	0
*LGALS9*	0.95 (0.918–0.983)	.003	0.948 (0.917–)	0

### 3.6 Clinical relevance of CD58, HAVCR2, and LGALS9 in 32 grade II and III patients

**Figure 6:**
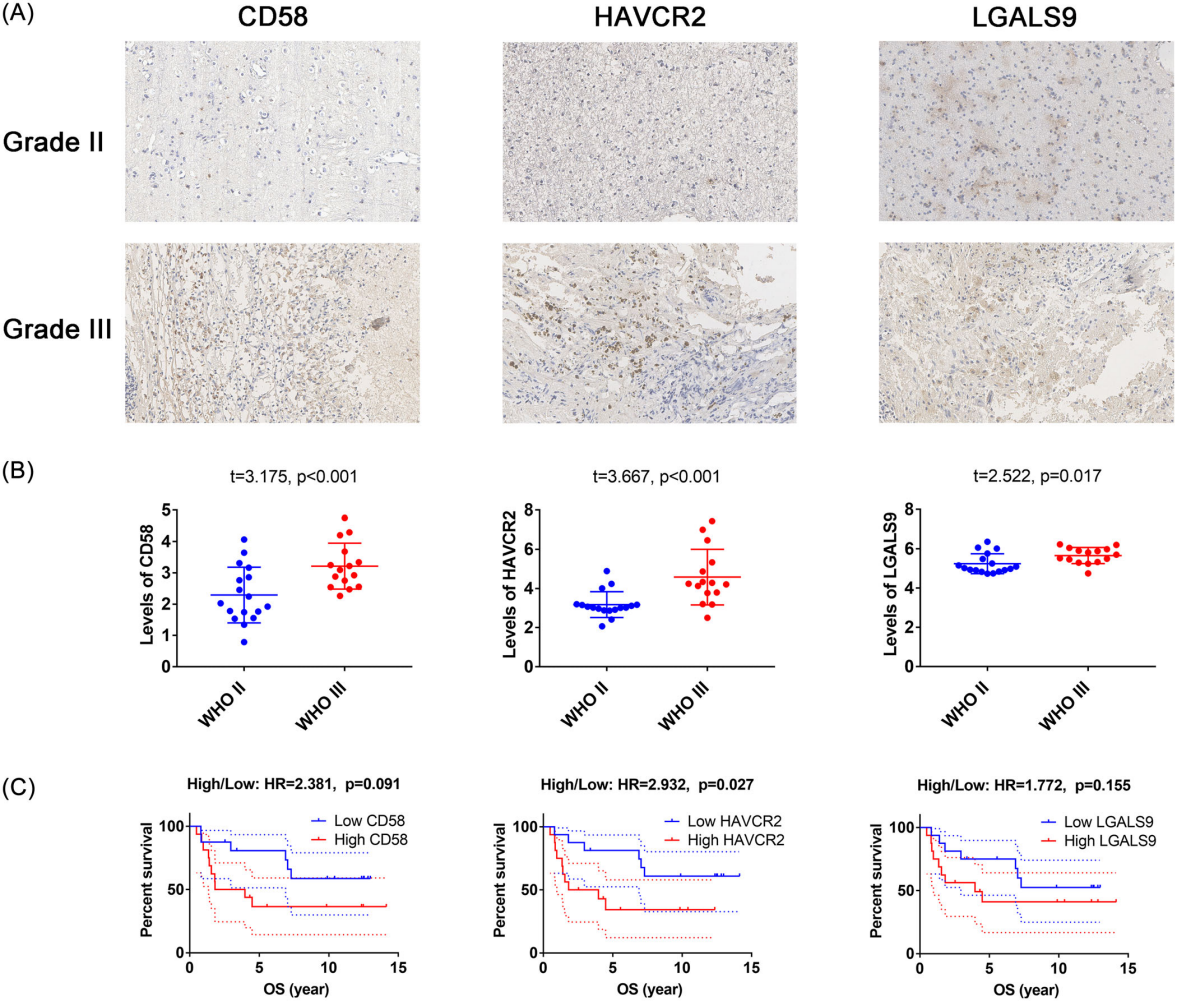
The levels of CD58, HAVCR2 and LGALS9 in 32 grade II and III patients. (A) Immunohistochemistry images of CD58, HAVCR2 and LGALS9 staining in the WHO grade II and III (×: 200 fold). (B) Immunohistochemistry H‐score of CD58, HAVCR2 and LGALS9 staining in the WHO grade II and III. (C) The relationship of CD58, HAVCR2 and LGALS9 with OS.

A total of 32 LGG patients (aged 50.34 ± 2.03 years, with a range of 26–69 years) were included in this study, including 17 grade II and 15 grade III cases. Immunohistochemistry (IHC) analysis was performed to measure the expression of CD58, HAVCR2, and LGALS9, as shown in Figure 6A. An unpaired *t*‐test revealed higher H‐scores of CD58, HAVCR2, and LGALS9 in WHO grade III compared to WHO grade II gliomas (*p* < .05), as shown in Figure 6B. GEPIA analysis showed that HAVCR2 expression was associated with poor OS in LGG patients (HR = 2.932, *p* = .027), as shown in Figure 6C.

## 4 DISCUSSION

Gliomas, the most common primary malignant brain tumors, present a therapeutic challenge due to their heterogeneity in clinical behavior.^13^, ^14^ While surgical resection and chemotherapy offer better prognosis, the emergence of targeted therapies has led to an increased interest in immunotherapy, tumor microenvironment, and combination approaches.^15^ In this study, we investigated the prognostic value of the CD2–CD58 axis in LGG patients using the TCGA database and an IHC experiment. Our results indicated that the CD2–CD58 axis is notably related to poor survival in LGG patients, and high CD58 expression contributes to T cell‐mediated immune responses by affecting inhibitory immune checkpoint genes.

The tumor immune microenvironment plays a crucial role in tumorigenesis, tumor progression, and recurrence.^16^ Cancer cells, stromal cells, immune cells, and their extracellular factors have profound effects on promoting or suppressing anticancer immunity.^17^ CD142 appears to be a master switch regulating the radiation response in glioblastoma patients, with striking effects on senescence‐like and mesenchymal tumor phenotypes as well as the microenvironment.^18^ Siglec‐9 acts as an immune‐checkpoint molecule on macrophages in glioblastoma, restricting T cell priming and immunotherapy responses in glioblastoma patients.^19^ Our study investigated the prognostic value of the CD2–CD58 axis in LGG patients. CD2, CD48, and CD58, closely related to the immunoglobulin superfamily, are involved in T cell responses to cancer cells.^20^ The disruption of the CD2–CD58 axis can lead to immune evasion, not only by impairing T cell co‐stimulation through CD2 but also by increasing T cell inhibition through PDL1‐PD1 signaling.^21^ Targeting CD2–CD58 interactions has therapeutic relevance in undesired immune responses.^22^ In vitro studies have shown that the deficiency of CD58 limits the recognition of tumor cells by T/NK cells, leading to immune evasion in a CD2/CD58‐dependent manner.^23^, ^24^

Furthermore, our study highlighted the critical role of the tumor immune microenvironment in glioma progression and recurrence. Our findings from the TCGA database and our cohort demonstrated that CD2–CD58 axis expression levels are associated with WHO grades in LGG patients. Cox's regression analysis identified CD58 as an independent prognostic marker for poor prognosis in LGG patients. Recent findings in the molecular landscape of adult LGG have refined the understanding of the heterogenous tumor populations. Notably, CD58 is implicated in immune cell infiltration in WHO grade II and III but not in WHO grade IV patients. CD58 enhances immune escape by affecting T cell activation and leukocyte‐mediated immunity, potentially contributing to poor prognosis in tumors.

The immune competence in cancer has a significant impact on the efficacy of conventional medical therapy. Immune checkpoint inhibitors, such as those targeting cytotoxic T lymphocyte‐associated molecule 4 (CTLA‐4), programmed cell death receptor 1 (PDCD1, also named PD‐1), and programmed cell death ligand 1 (CD274, also named PD‐L1), have revolutionized cancer treatment.^25^ Oncologists have given a great deal of attention to the anticancer immune responses for the successful treatment in many cancers.^26^ However, a substantial proportion of patients show refractoriness to these inhibitors. Other immune checkpoints, including LAG‐3, TIM‐3 (also known as HAVCR2), TIGIT, and VISTA, have emerged as promising targets in cancer immunotherapy.^27^

Additionally, our study demonstrated a close association between CD58 and the TIM‐3/GAL9 immune checkpoint, rather than CTLA‐4 or PD‐1/PDL‐1 signaling, in LGG patients. Uni‐variable and multi‐variable logistic analyses identified TIM‐3 and GAL9 as independent risk factors for poor prognosis in LGG patients. IHC experiments supported the correlation between TIM‐3 expression (measured by H‐score) and poor prognosis. We speculate that the disruption of the CD2–CD58–TIM‐3 axis contributes to immune evasion in LGG patients, potentially leading to intrinsic resistance to CAR T cell therapy. This finding highlights the heterogeneity within LGGs and suggests that CD58 may play a role in immune escape mechanisms and contribute to poor prognosis in these tumors. Understanding the immune landscape and the interactions between tumor cells and immune cells is crucial for developing effective immunotherapeutic strategies tailored to individual patients. These findings expand the repertoire of immune checkpoints that can be targeted to enhance anticancer immune responses and overcome treatment resistance in LGGs patients.

In summary, immune checkpoint modulators have become integral to immunotherapy in the field of oncology. Our study highlights the CD2–CD58 axis‐related immune signature as a valuable prognostic biomarker in LGG patients, with increased CD58 expression potentially serving as an immune evasion mechanism. Considering the heterogeneity across patients and WHO grades, individualized combination strategies targeting TIM‐3/GAL9 could enhance the efficacy of immunomodulatory approaches and overcome treatment resistance in LGG patients.

## AUTHOR CONTRIBUTIONS

**mingwei Wu**: Data curation; Resources; Writing—original draft. **yiyuan Chen**: Formal analysis; Methodology. **Gao Hua**: Investigation; Validation. **Liu Chunhui**: Project administration; Writing—review & editing.

## CONFLICT OF INTEREST STATEMENT

The authors declare no conflict of interest.

## Data Availability

All the data generated or analyzed in this study are included in this published article and its additional files. This study was conducted according to the guidelines of the Declaration of Helsinki and approved by the Institutional Review Board Beijing of Tiantan Hospital, affiliated with the Capital Medical University (No. KY2016‐035‐01).
